# Rapid seagrass meadow expansion in an Indian Ocean bright spot

**DOI:** 10.1038/s41598-024-61088-1

**Published:** 2024-05-13

**Authors:** Matthew Floyd, Holly K. East, Dimosthenis Traganos, Azim Musthag, James Guest, Aminath S. Hashim, Vivienne Evans, Stephanie Helber, Richard K. F. Unsworth, Andrew J. Suggitt

**Affiliations:** 1 Department of Geography and Environmental Sciences, Faculty of Engineering and Environment, Northumbria University, Newcastle Upon Tyne, NE1 8ST UK; 2 German Aerospace Centre (DLR), Remote Sensing Technology Institute, 12489 Berlin, Germany; 3 Small Island Research Group, Faresmaathoda, 10780 Maldives; 4 School of Natural and Environmental Sciences, Newcastle University, Newcastle Upon Tyne, NE1 7RU UK; 5 Blue Marine Foundation, M. Beach Side, Handhuvaree Hingun, Malé, 20285 Maldives; 6 Blue Marine Foundation, Somerset House, Strand, London, WC2R 1LA UK; 7 Seagrass Ecosystem Research Group, Faculty of Science and Engineering, Swansea University, Swansea, SA2 8PP Wales, UK

**Keywords:** Marine biology, Tropical ecology, Ecology, Conservation biology, Climate-change ecology

## Abstract

The areal extent of seagrass meadows is in rapid global decline, yet they provide highly valuable societal benefits. However, their conservation is hindered by data gaps on current and historic spatial extents. Here, we outline an approach for national-scale seagrass mapping and monitoring using an open-source platform (Google Earth Engine) and freely available satellite data (Landsat, Sentinel-2) that can be readily applied in other countries globally. Specifically, we map contemporary (2021) and historical (2000–2021; n = 10 maps) shallow water seagrass extent across the Maldives. We found contemporary Maldivian seagrass extent was ~ 105 km^2^ (overall accuracy = 82.04%) and, notably, that seagrass area increased threefold between 2000 and 2021 (linear model, + 4.6 km^2^ year^−1^, r^2^ = 0.93, p < 0.001). There was a strongly significant association between seagrass and anthropogenic activity (p < 0.001) that we hypothesize to be driven by nutrient loading and/or altered sediment dynamics (from large scale land reclamation), which would represent a beneficial anthropogenic influence on Maldivian seagrass meadows. National-scale tropical seagrass expansion is unique against the backdrop of global seagrass decline and we therefore highlight the Maldives as a rare global seagrass ‘bright spot’ highly worthy of increased attention across scientific, commercial, and conservation policy contexts.

## Introduction

Seagrass meadows form foundational habitats across the globe that mitigate the impacts of climate change and make key contributions towards the achievement of Sustainable Development Goals (SDGs) in coastal areas^1^. For example, seagrasses supply nursery habitat for 20% of the world's most productive fisheries^2^; reduce marine bacterial pathogens by 50%^3^; buffer wave energy reaching coasts by 40%^4^; and supply sediment that maintains reef island shorelines^5^. Additionally, despite only occupying some ~ 0.1% of the seafloor, larger and persistent seagrass species contribute to sequestering up to 18% of the global oceanic carbon in their sediments and are more efficient at storing CO_2_ than tropical forests^6^. Referred to as ‘blue carbon’ systems, these habitats have been identified as a conservation priority to mitigate climate change impacts.

Despite their highly valuable societal benefits, seagrasses are facing a crisis globally^7^. Seagrass habitats are exposed to a range of direct threats, including declining water quality, coastal development, and destructive fishing practices^8^; and indirect regional and global climate change threats^9^, such as heatwave events^10^ and increases in storm frequency and severity^11^. As a result of these co-occurring stressors, seagrass area decline is estimated globally at a rate of 1–2% per year with some regions experiencing losses of ~ 7% per year, which equates to the area of one football field every 30 minutes^12^. The degradation of seagrass meadows is of global concern because it inevitably leads to the associated loss of both their societal benefits and wider ecosystem functioning^10,13^. Therefore, the need to conserve and restore seagrass systems is urgent as there is still time to facilitate the recovery of seagrass habitat functions and services^14^.

Continued monitoring of seagrass is important to measure the benefits of seagrass habitat, and to inform marine management and conservation. Yet a significant obstacle to monitoring and, in turn, management decisions, is a lack of spatial information on seagrass extent/distribution^15^. Global analyses of seagrass area trajectories show that, although efforts have been made to address existing data gaps ^16–18^, many areas of South America, east Africa, the Indo-Pacific, and the central Indian Ocean lack spatially-explicit information on seagrass beds^7^. Furthermore, modelling shows areas with the highest potential for seagrass decline often lack spatially explicit data^19^.

Cloud-based satellite mapping is an effective approach to fill these data gaps and has been used to produce global-scale geomorphic and benthic cover maps as part of the Allen Coral Atlas project^18,20^ and bioregional seagrass maps for the Mediterranean basin^17^. A limited number of studies have demonstrated the ability to map seagrass change over time using Landsat imagery, mainly over limited spatial scales and/or at reduced temporal resolution^21–23^. Upscaling workflows over larger spatial scales and at higher temporal resolution is a clear next step in seagrass habitat mapping and could provide rich information on regional and national seagrass trajectories to inform data-driven seagrass conservation.

Here, we demonstrate a novel workflow that uses a freely available cloud-based platform (Google Earth Engine) and freely available satellite data to map and quantify shifts in the areal extent of seagrass meadows. We apply this workflow across the Maldives Archipelago, which offers an especially interesting location to analyse national-scale temporal changes in seagrass meadows because (1) there is a paucity of high-confidence seagrass data from this nation^24^; (2) societal attitudes towards seagrass in the Maldives can be negative whereby seagrass is often viewed as a nuisance^25^; (3) resorts in the Maldives, and increasingly local islands with small scale tourist facilities, actively and routinely remove seagrass habitat for aesthetic purposes^25^; and (4) planning for a series of inshore protected areas could include seagrass habitat if thematic maps are produced (see www.nooraajje.org). Specifically, the objectives were to: (1) create a contemporary (2021) national-scale shallow seagrass map for the Maldives; (2) create a series of historical national-scale seagrass maps for the Maldives between 2000 and 2021; (3) use the series of seagrass maps produced in (2) to quantify shifts in seagrass area and distribution between 2000 and 2021; and (4) analyse the relationships between seagrass area and potential controlling variables, including abiotic environmental conditions, land use, and population changes^26,27^. Our approach will provide spatial seagrass data that can be fed into marine spatial planning frameworks and inform protected area designations in the Maldives.

## Results

### National scale contemporary seagrass map

**Figure 1:**
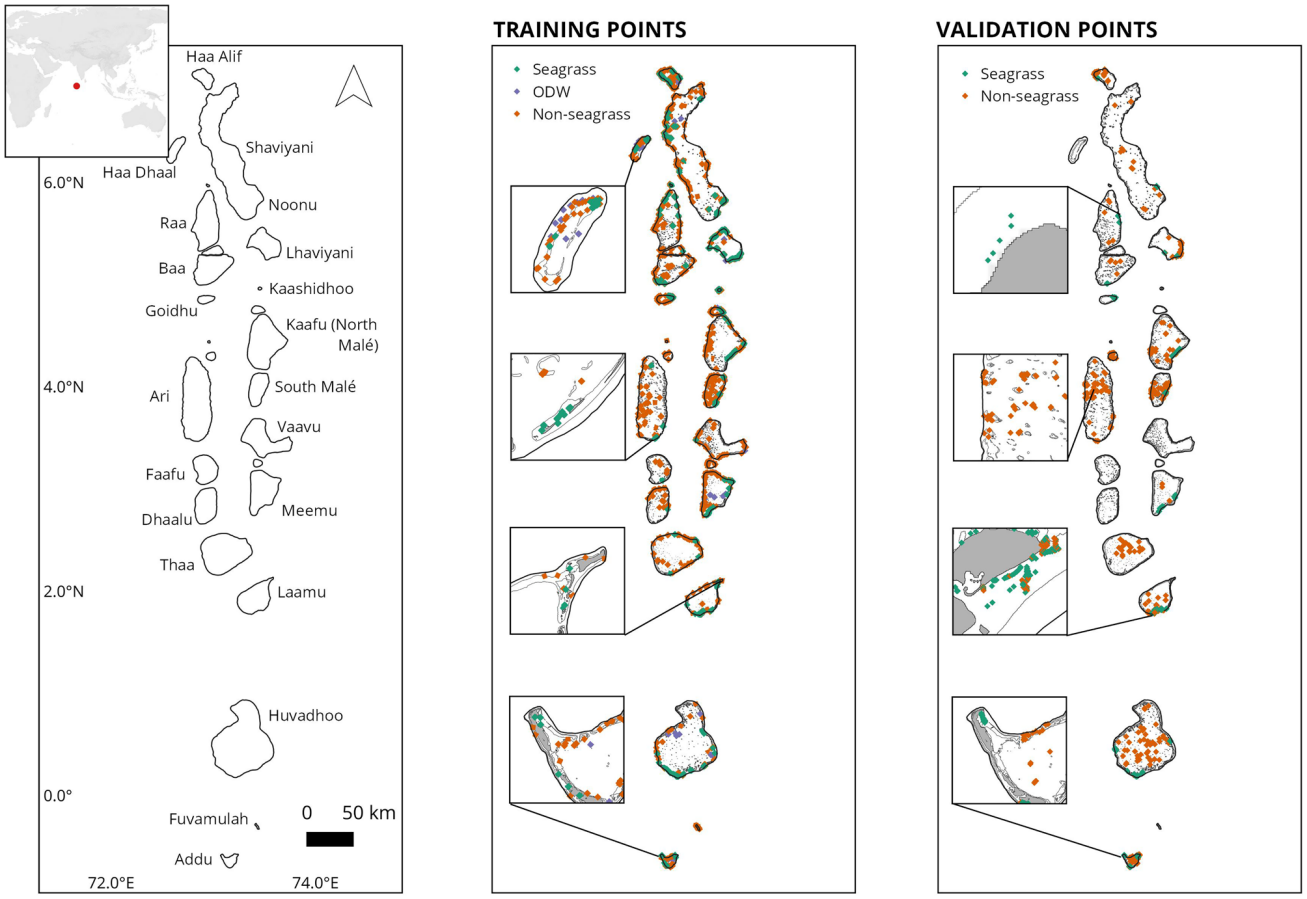
Left: The Maldives archipelago atoll outlines and atoll names; Middle: distribution of machine learning classifier training points labelled manually through photointerpretation (n = 5761 seagrass; n = 15,433 non-seagrass; n = 4269 Optically Deep Water); Right: distribution of seagrass map validation points (n = 557 seagrass; n = 462 non-seagrass).

**Figure 2:**
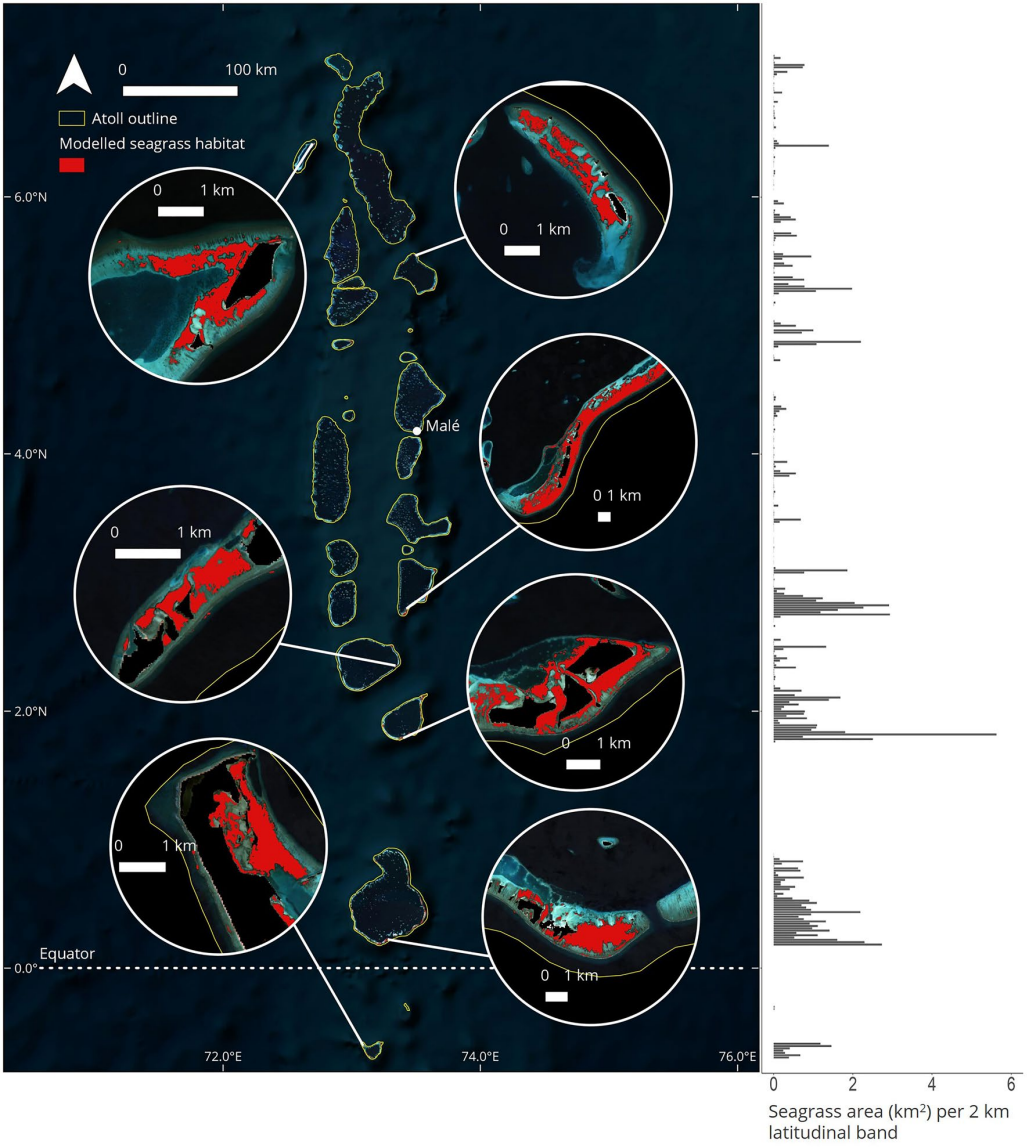
Left: Maldivian seagrass habitat extent modelled in 2021 using Sentinel-2 data with SVM classification; Right: Sum of seagrass area in latitudinal bands of 2 km width, plotted across the full Maldives extent.

The workflow presented used data from 881 Sentinel-2 level-1C TOA images spanning 01.01.2021–31.12.2021 to generate a composite image over an area of 22,840 km^2^ (Fig. 1). The resulting binary seagrass classification from the SVM model identified 105 km^2^ of shallow water seagrass habitat across the Maldives in 2021 with an overall accuracy of 82.04% (seagrass producer accuracy = 74.15%; seagrass user accuracy = 91.37%) (Fig. 2).

The model identified a substantial proportion of seagrass habitat in the south, with 76% (79.8 km^2^) of the seagrass area found south of the latitudinal centre of the country (the Maldives archipelago extends from 0.7°S to 7°N). Seagrass extent was also unevenly distributed across atolls, with 67% of the total seagrass area present on 3 atolls (Huvadhoo = 29.3 km^2^; Laamu = 21.9 km^2^; Meemu = 19.4 km^2^) (Supplementary Table 1). The largest continuous seagrass meadow was 12.8 km^2^, which was located on the atoll rim adjacent to Kolhufushi (Meemu atoll).

### National scale historical seagrass maps

The workflow used to produce historical seagrass maps for the Maldives retrieved 1,689 Landsat scenes (1,261 Landsat 8; 428 Landsat 7) between 2000 and 2021 (Table 1). Accuracy assessments for classification products showed high accuracy across all years (> 89% in all cases; Table 2).

**Table 1:** Maldivian multi-annual composite scenes, satellite source, and total number of training pixels used to inform an SVM classifier of seagrass extent.

Class year	Number of scenes	Satellite	Training pixel sum		
			Seagrass	Non-seagrass	ODW
2020–2021	311	L8	747	2477	510
2018–2019	292	L8	855	3259	726
2016–2017	331	L8	941	3308	581
2014–2015	327	L8	818	3331	581
2012–2013	93	L7	827	3285	725
2008–2009	70	L7	861	3692	676
2006–2007	67	L7	690	3611	821
2004–2005	80	L7	913	3643	794
2002–2003	54	L7	894	3637	810
2000–2001	64	L7	922	3640	777

**Table 2:** Accuracy assessment results for Maldivian seagrass habitat maps. Data from Landsat time series and Sentinel-2 satellite archives.

	Landsat historical maps										Sentinel-2 contemporary map
	2000–2001	2002–2003	2004–2005	2006–2007	2008–2009	2012–2013	2014–2015	2016–2017	2018–2019	2020–2021	2021
Overall accuracy	89.1	89.6	92.6	91.6	93.3	94.1	93.1	94.4	95.3	90.3	82.04
Producer accuracy non-seagrass	99.0	98.3	93.8	97.0	97.3	94.8	96.0	95.0	98.3	94.0	91.56
Producer accuracy seagrass	79.3	81.0	91.5	86.3	89.3	93.5	90.3	93.8	92.3	86.5	74.15
User accuracy non-seagrass	82.7	83.8	91.7	87.6	90.0	93.6	90.8	93.8	92.7	87.4	74.60
User accuracy seagrass	98.8	97.9	93.6	96.6	97.0	94.7	95.8	94.9	98.1	93.5	91.37

**Figure 3:**
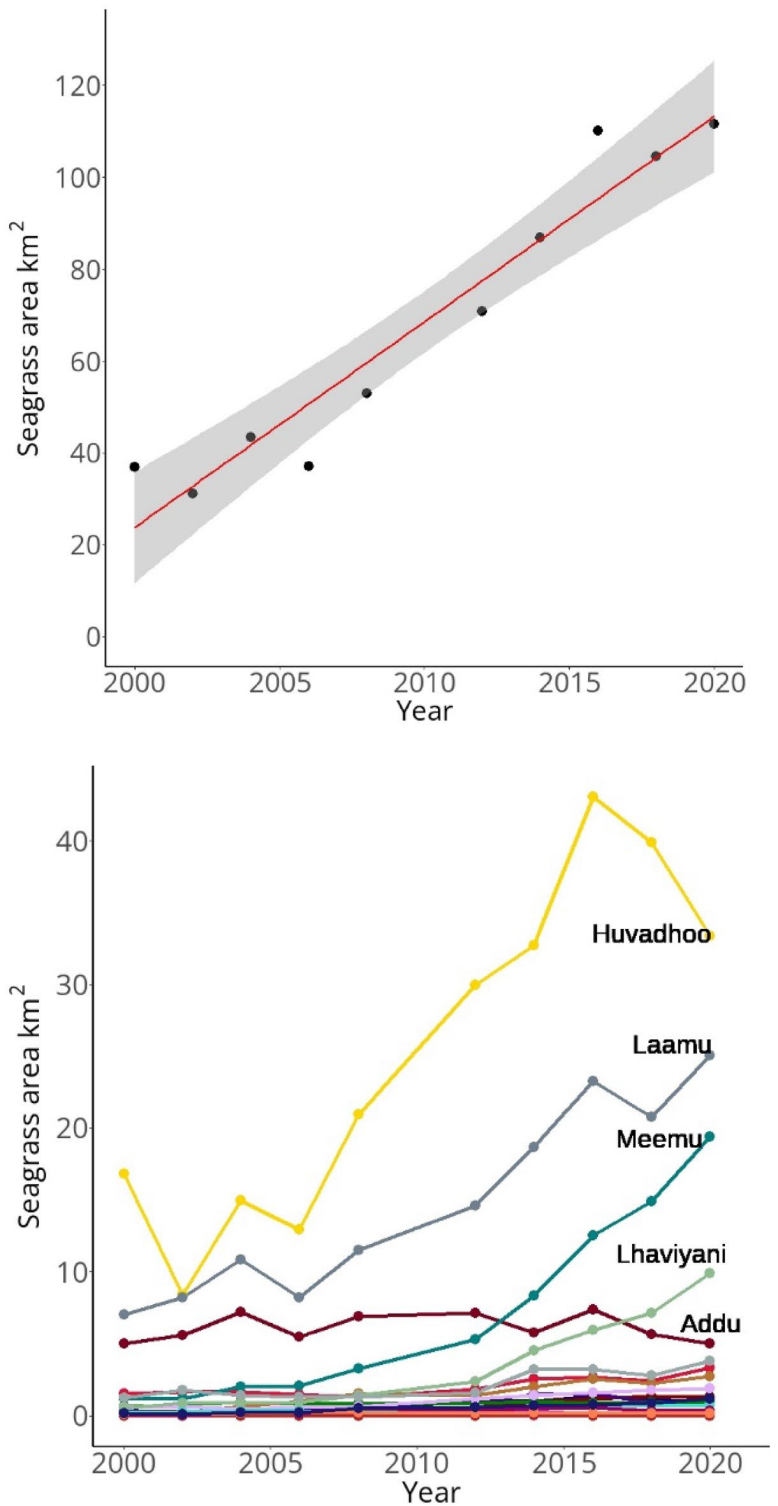
Top: Maldivian seagrass habitat area change from 2000 to 2021 (r^2^ = 0.93); Bottom: Maldivian seagrass area change across 26 atolls from 2000 to 2021. Data from Landsat 7 and 8 archives and seagrass habitat modelled using SVM classification.

**Figure 4:**
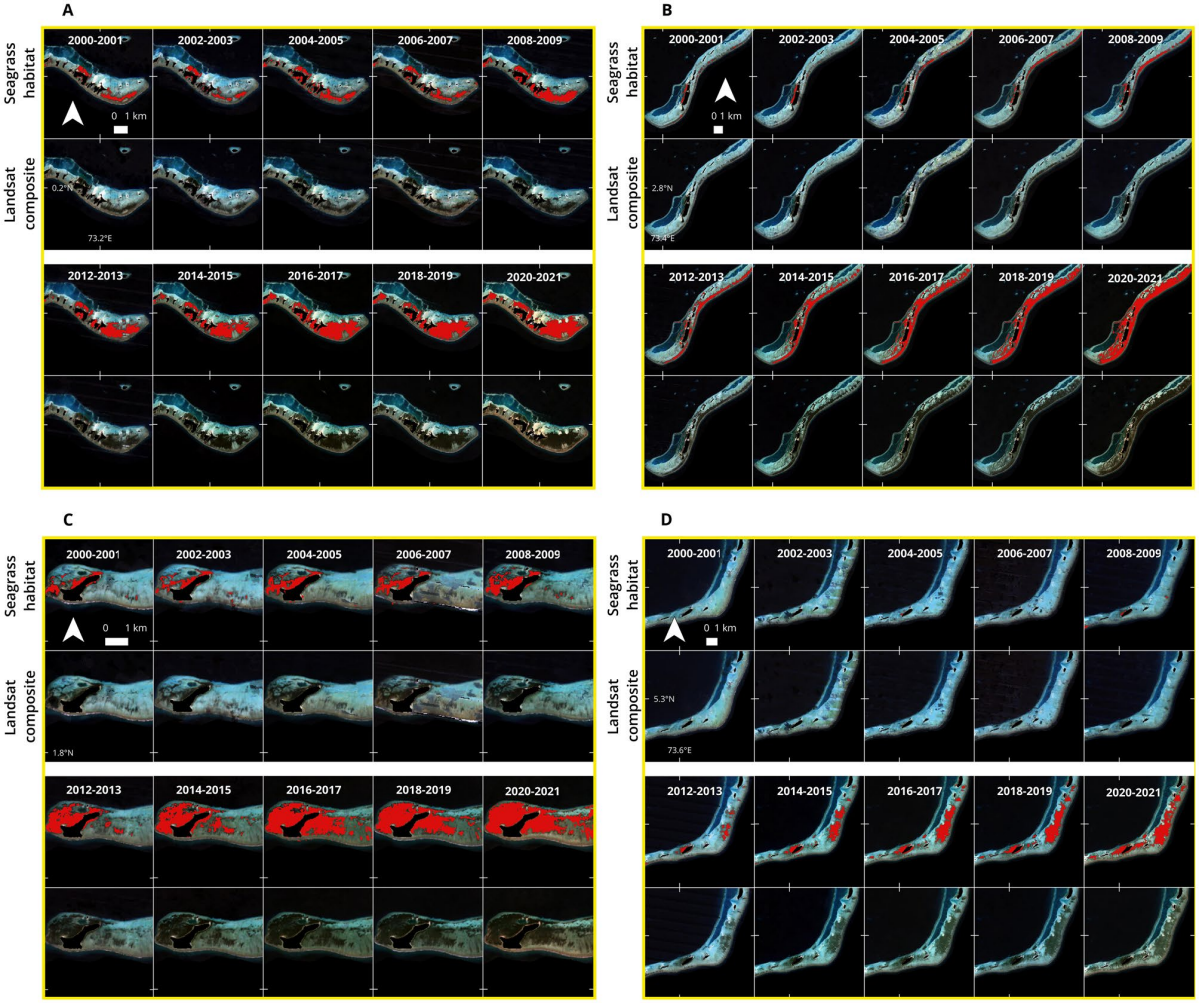
Seagrass habitat area expansion from 4 example sites in the Maldives between 2000 and 2021. Satellite data are from the Landsat data archive and seagrass habitat area is predicted from an SVM classifier. (**A)**: Faresmaathoda, Huvadhoo atoll; (**B**): Kolhufushi, Meemu atoll; (**C)**: Gaadhoo, Laamu atoll; (**D)**: Olhuvelifushi, Lhaviyani atoll.

Notably, there was a highly significant increase in seagrass areal extent through time, whereby seagrass area increased threefold from 37 km^2^ in 2000–2001 to 111 km^2^ in 2020–2021 (Figs. 3 and 4; + 4.6 km^2^ year^−1^; r^2^ = 0.93; F = 125.9; DF = 1,8; p < 0.001). Increases in seagrass area between 2000 and 2021 were greatest on Meemu (+ 18.2 km^2^; percentage change =  + 1,499%), Laamu (+ 18.1 km^2^; percentage change =  + 256%), Huvadhoo (+ 16.5 km^2^; percentage change =  + 97%), and Lhaviyani (9.4 km^2^; percentage change =  + 1,680%) atolls (Fig. 3). The increases on these 4 atolls represented 83% of the total increases in seagrass area across the Maldives.

There were moderate seagrass area gains between 2000 and 2021 on Shaviyani (+ 2.6 km^2^; percentage change =  + 204%), Goidhu (+ 2.5 km^2^; percentage change =  + 1,018%), Thaa (+ 1.8 km^2^; percentage change =  + 116%), Kaashidhoo (+ 1.3 km^2^; percentage change =  + 225%), Haa Dhaal (+ 1.0 km^2^; percentage change =  + 384%), and South Malé (+ 1.0 km^2^; percentage change =  + 347%) atolls. On Addu, the most southerly atoll, seagrass area was stable over time (2000–2001 area 5.0 km^2^; 2020–2021 area 5.04 km^2^).

None of the atolls showed substantial overall losses of seagrass area between 2000 and 2021, the largest loss (albeit moderate) was recorded on Dhaalu (− 0.2 km^2^; percentage change = − 89%). However, despite exhibiting the largest seagrass extent in 2020–2021, the seagrass area on Huvadhoo experienced declines later in the time series, reducing by 9.7 km^2^ from a peak of 43.1 km^2^ in 2016–2017 (Fig. 3).

### Analyses of the controls on seagrass distribution

Logistic regression of the 2021 data revealed significant effects of both abiotic environmental and anthropogenic factors on seagrass presence, with the best model accounting for 20% of the variation in seagrass presence/absence across the Maldives (McFadden pseudo r^2^ = 0.20). The model showed an increased likelihood of seagrass presence in response to human habitation. On average, with all other variables held constant, the chance of seagrass presence on inhabited reef platforms—shallow coral reef derived geomorphic features that can support reef islands—in 2021 was 344% greater than on uninhabited reef platforms (p < 0.001). There was no significant effect of population density on the chance of seagrass presence. Two abiotic factors—mean depth and mean ruggedness – also had a significant effect on seagrass presence. There was an increased probability of seagrass presence on reef platforms with a shallower mean depth, on average the chance of presence increased by 0.19% for every 1-m shallowing of depth (p < 0.001). The model also showed that for every one-unit increase in ruggedness index, the chance of seagrass presence decreased by 0.40% (p < 0.001).

**Figure 5:**
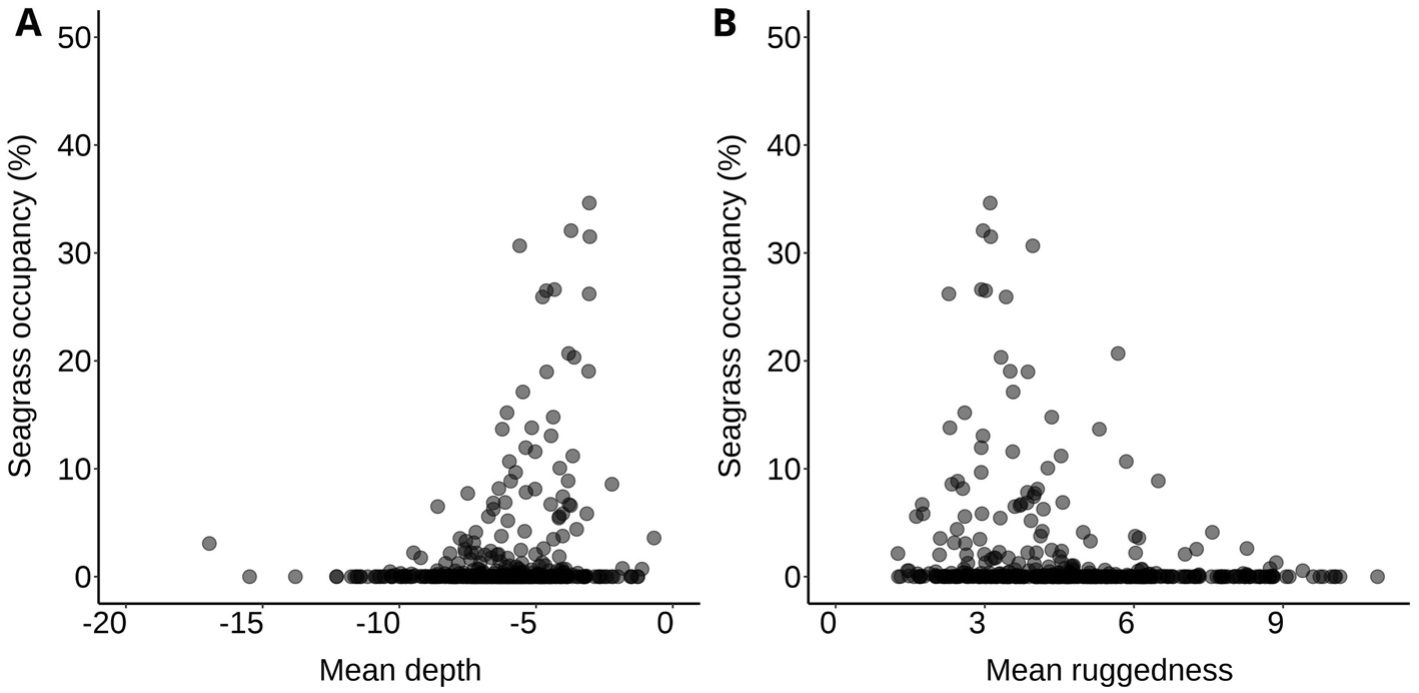
Relationship between seagrass occupancy (%) and 2 environmental predictor variables (**A**) mean depth (metres) and (**B**) mean ruggedness from shallow reef platforms in the Maldives. Seagrass occupancy data were from an SVM classifier of Sentinel-2 imagery.

The generalised linear model of seagrass occupancy accounted for 13% of the variation in the response variable and found significant effects of environmental factors and land use (McFadden pseudo r^2^ = 0.13). The model showed occupancy of seagrass habitat was positively associated with shallower reef platforms and increased by 1.18% with each 1-m reduction in depth (Fig. 5; p < 0.05). Furthermore, increases in the mean ruggedness of reef platforms were also associated with a lower expected occupancy of seagrasses, on average, whereby for each unit increase in the ruggedness of reef platforms seagrass occupancy decreased by 1.99% (Fig. 5; p < 0.001).

**Figure 6:**
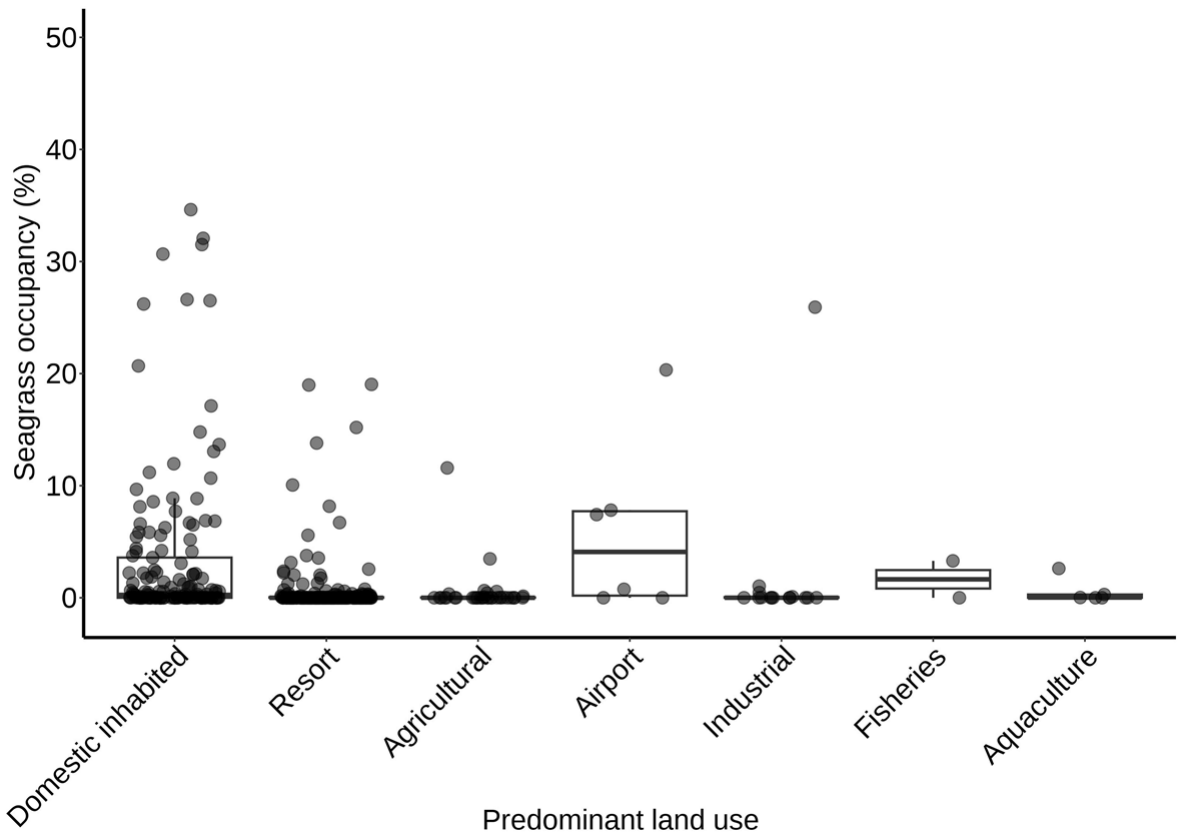
Seagrass occupancy from inhabited Maldivian reef platforms with 7 different predominant land use types. Seagrass occupancy data were from an SVM classifier of Sentinel-2 imagery.

The seagrass occupancy model also found significant differences in seagrass occupancy between land use types (Fig. 6). When compared to domestic inhabited islands, there was significantly lower seagrass occupancy on reef platforms with predominantly agricultural (p < 0.001) and industrial (p < 0.05) islands (Fig. 6). Seagrass occupancy did not differ significantly between resort islands and domestic islands.

The generalised linear Poisson model used to explore seagrass gains on inhabited reef platforms showed no significant effect of population increase over the time series (p = 0.502).

## Discussion

Here we present a unique multi-decade analysis of seagrass showing globally significant increases in areal extent that contrast with projections of continued decline across many bioregions^12,14^. Using cloud computing, satellite imagery, and field data we also present the first high-confidence seagrass-specific maps for the Maldives over a study area of 22,840 km^2^. The contemporary seagrass map estimates that the shallow water seagrass area in the Maldives was 105 km^2^ in 2021. Through generating a time series of national seagrass maps (n = 10 time points) we found a threefold increase in seagrass area between 2000 and 2021. Tropical seagrass areal expansion at the national scale is (to the best of the authors’ knowledge) a globally unique situation. Seagrass meadows in the Maldives have previously been underappreciated and there is a near-absence of published data on Maldivian seagrasses^25^. Here, we wish to highlight the Maldives as a seagrass bright spot, whereby national-scale seagrass expansion is in stark contrast to reported global declines^7,12^. Our data also show a higher probability of seagrass presence on reef platforms with inhabited islands (vs uninhabited islands), and the greatest expected occupancy of seagrass beds on reef platforms with inhabited and resort islands (vs agricultural and industrial land use types), shallower water depths, and lower ruggedness. We thus hypothesize that seagrass expansion is driven by modest anthropogenic influence on the marine environment.

### Contemporary Maldivian seagrass extent

We estimate that seagrass habitat accounted for 3.63% of shallow benthic habitat cover in the Maldives in 2021^20^, making seagrass the largest blue carbon system by area in the Maldives. Currently, data on wetlands and mangrove areas are incomplete, but estimates from 74 islands suggest a total area of 7.39 km^2,26,28^, equal to just 7.04% of the 2021 seagrass area. Our estimates also expand upon previous mapping efforts that include the Maldives by identifying a further 62.72 km^2^ of seagrass habitat^20^ (seagrass estimate for Maldives 42.28 km^2^; accessed August 2023^20^).

Seagrasses in the Maldives are a crucial part of the tropical seascape and, as such, accurate accounting of seagrass habitat is important to conserve and quantify the value of their associated coastal ecosystem services. For example, data on seagrass habitat area can be used to upscale estimates of in-situ ecosystem services, such as blue carbon storage^16^ and sediment production^5^. However, it is also important to note that, particularly for seagrass blue carbon, extensive in-situ data collection is required to fully characterise the high spatial and temporal variability of carbon stocks^29,30^. It is currently estimated that ~ 17% of Indo-Pacific seagrasses are included in MPAs, which represents the smallest proportion of protected seagrass area of all seagrass bioregions^15^. We suggest that the findings of this study can support a range of marine conservation initiatives in the Maldives, which can directly and indirectly contribute to achieving international conservation and climate change mitigation targets^15^.

Our results showed a positive association between seagrass presence and shallower water depths (within the 0–20 m scope of our study), an unsurprising result given that depth is a fundamentally important variable that governs the lower vertical limits of seagrasses globally^31^. As photoautotrophs, seagrasses are limited by light availability in the photic zone of the water column which attenuates with increasing depth. Interestingly, seagrass beds are also associated with localised shallowing of water depth through sediment elevation^32^ which could contribute to the observed increased probability of seagrass presence in shallower waters. Furthermore, a positive feedback mechanism may exist, whereby the shallowing of water depth through sediment elevation benefits seagrass by increasing the availability of light. Therefore, it is likely that the association between seagrasses and shallower waters is a product of two processes: (1) increased light availability providing good growth conditions in shallow waters, and (2) seagrass beds reducing localised water depths through sediment accretion.

Lower mean ruggedness was also associated with increased presence and occupancy of seagrasses. Shallow sites with low ruggedness scores may likely be more representative of sandy regions, whereas high ruggedness scores may represent regions with coral-derived reef structures and channels. As such, our results confirm the expected distribution of seagrass in sandy flat regions. Increased deposition and production of sediments associated with seagrasses^5,32^ could also contribute to the association between ruggedness scores and seagrass presence reported here by lowering the average ruggedness of seagrass sites.

### Seagrass expansion

Seagrass meadows have shown evidence of consistent declines over the last century across the Indo-Pacific and globally^7^. As such, Maldivian seagrass habitat trajectories are unique in their expansion over the last 20 years (Fig. 3). Although we lack long term seagrass habitat baselines from the Maldives, aerial imagery across a limited number of atolls shows low seagrass extent on reef platforms as far back as 1969 ^26^. Whilst we cannot draw conclusions from these data, they could suggest the trend between 2000 and 2021 can be contextualised as part of a longer-term expansion (up to 50 years)^26^. Ultimately, missing baseline data preclude us from confidently inferring the trends in seagrass extent prior to our study timescale and we cannot comment on seagrass extent prior to 1969. Nevertheless, the success of seagrasses contrasts with substantial declines in Maldivian coral reef health^33,34^ and recent mangrove die-offs (Carruthers et al., unpublished data). The connectivity between corals, mangroves, and seagrasses in the Indo-Pacific is strong^35^. Therefore, the ecosystem functions of healthy seagrass beds, such as the provision of nursery grounds for juvenile fishes^36^, improvements in water quality^37^, reductions of localised seawater acidity^38^, and reduction of marine pathogens^3^, may play an important supportive role in buffering pressures on coral and mangrove systems.

Given our results show a strong association between habitation and seagrass presence, we hypothesize that seagrass expansion is driven by anthropogenic activity in the Maldives. Previous work from the central Maldivian atolls shows that the human population and associated activities influence the benthic composition of reefs^39^. It is possible that nutrient inputs from anthropogenic sources could lead to the enrichment of nitrogen and/or phosphorous in the coastal zone, as has been shown previously in the Maldives^27^, contributing to the expansion of seagrass^40^. We suggest that nutrient levels are below a critical threshold that would tip the seagrass habitats into eutrophic conditions with negative implications for seagrass health. Currently, it may be that the naturally low nutrient oceanic waters^41^, coupled with high advective flushing of reef platforms limit the flux of nutrients into the coastal system^42^. Indeed, seagrass growth and production, particularly at the seedling stage, is largely limited by the availability of nutrients in temperate and tropical regions and moderate increases in nutrient concentrations have been shown to stimulate their production and growth^43,44^. Nutrient sources include sewage, domestic waste, fertilisers, and wastewater that can enter the marine environment through surface water runoff and/ or groundwater discharge through the porous reef island sediments^42,45,46^. In many cases, appropriate waste management is limited in the Maldives, and contamination of groundwater from anthropogenic waste is recorded on many inhabited islands across the country^26,45^.

In addition to nutrient enrichment, artificial alteration of sediment dynamics through nationwide anthropogenic island development could also contribute to seagrass expansion. For example, during land reclamation and dredging activities, suspended sediment concentrations in the water column can increase locally^47,48^. Such activities are common on inhabited Maldivian islands to develop infrastructure and to expand habitable land for the growing human population^25,26^. As seagrasses promote the settlement of particulate matter^32^, the suspended sediments from these common and widespread anthropogenic activities could preferentially settle in seagrass habitats and persist following the cessation of the disturbance. This process could improve localised growing conditions for seagrasses by providing suitable sediment habitat through accretion that will also reduce localised water depths and in turn provide improved light availability in the medium to long term.

Whilst seagrass expansion may be an advantage of anthropogenic activity, if certain trends continue, seagrass habitat could suffer declines in health and extent. For example, if nutrient levels continue to increase, there could be a shift to conditions favouring macroalgal growth^49^. Macroalgal growth is strongly associated with declines in both seagrass and coral growth as it blocks sunlight required for photosynthesis and creates unfavourable biogeochemical conditions^50,51^. In coral reef systems, the proliferation of macroalgae can result in a shift from ‘hard reef’ (coral-dominated) to ‘soft reef’ (algal-dominated) states, as has been documented in the Caribbean^52,53^. Such coral-macroalgal phase shifts can cause the ecological collapse of reef systems as they may induce a shift to an alternate ecological state dominated by a different suite of organisms^54^, a loss of habitat complexity^55^, a transition from a state of net reef accretion to net erosion^56^, and a reduction in reef resilience to disturbance events^57^. As an atoll nation that is so dependent on the ecosystem services provided by coral reefs and seagrasses (the provision of habitable land, fisheries, and tourism), any coral-macroalgal phase shifts would have severe socioeconomic and ecological impacts on the Maldives.

On reef platforms adjacent to agricultural islands, trends of increasing fertiliser use may already lead to eutrophication in the coastal zone^26,42,45^. Indeed, our results showed that occupancy of seagrass habitat was lower adjacent to agricultural islands compared to inhabited and resort islands (Fig. 6). A potential explanation for this, although not tested here, could be that the nutrient enrichment from agricultural islands is above a threshold that causes detrimental effects on seagrasses^49^. Additionally, the use of fertilisers and pesticides is unregulated in the Maldives and therefore any potentially negative effects of these chemicals on the marine environment are largely unknown. Increased urbanisation, population growth, and unregulated fertiliser use across the country could lead to more widespread nutrient loading into coastal systems^26,42,45^. For example, waste production in the Maldives increased by 56% between 2006 and 2016^45^ and there have been reports of red tides and mass fish mortalities in 2007, 2008, and 2012 which could be indicative of eutrophication^26,58^. Furthermore, it is not clear how susceptible seagrasses and coral reefs are in the Maldives to nutrient inputs or how much protection can be provided by oceanic flushing.

Despite the ostensible success of Maldivian seagrasses, the habitat also shows limitations in spatial distribution across the country. Firstly, 83% of seagrass expansion was limited to just 4 atolls whereas the other 22 atolls showed either moderate gains or stable but small seagrass area (Fig. 3). Furthermore, 76% of the overall contemporary seagrass area was present in the southern half of the Maldives (Fig. 2). Although not directly explored here, north–south environmental gradients will likely influence the distribution of seagrass in the Maldives. For example, the more continuous nature of southerly atoll rims and a greater proportion of rims with islands^59^ increases the amount of suitable habitat with anthropogenic influence which could lead to greater seagrass occupancy. Ultimately, the limited spatial distribution of seagrass across few atolls and the concentration of habitat in the south may highlight that the habitat could still be considered vulnerable to future degradation. For example, Addu atoll in the south showed a consistent seagrass bed area of ~ 5 km^2^ across the time series. However, it is likely, based on observations of high-resolution Planet data^60^ and local planning reports, that dredging work beginning in 2023 has recently reduced the overall seagrass area as part of a project to reclaim ~ 1.93 km^2^ of land. We also found evidence of seagrass area decline on Huvadhoo atoll since 2016 (Fig. 3) which should be monitored closely over the coming years. As the Maldivian population continues to grow, it is likely that the need for space will become increasingly important and may lead to conflict between seagrass protection and the requirement for land.

Working to understand and change societal attitudes towards seagrass is a challenge for global seagrass conservation^61^ and is likely to become more important in the Maldives as local islands and resorts adapt to increases in seagrass area. For example, local Maldivian attitudes to seagrass bed expansion can be negative when the habitat interferes with boat mooring or ease of access to islands^25^. Furthermore, many resorts in the Maldives actively remove seagrasses to achieve aesthetic preferences for white sandy lagoons and beaches^25,26^. Our results indicate a lower seagrass occupancy on reef platforms with resorts than those with inhabited islands (Fig. 6), which could be related to active seagrass removal by the tourism industry. However, seagrass removal is largely unreported and therefore represents a knowledge gap. Currently, at the national scale, this removal has not resulted in widespread seagrass decline, however, as tourism is the largest and fastest-growing industry in the Maldives, accounting for 21.4% of GDP in 2021^62^, environmental initiatives ought to work collaboratively with the tourism sector to manage its impact on the local environment. There is hope that attitudes towards seagrass in the Maldives can be altered, for example, in 2019 a campaign by Blue Marine Foundation and Maldives Underwater Initiative coincided with voluntary protection of ~ 0.83 km^2^ of seagrass on resort islands^63^. Overall, seagrass expansion and high occupancy around domestic and resort islands can provide many benefits to local communities, but it is also important to consider the potential for conflict associated with the proliferation of this habitat.

To analyse national scale shifts in the areal extent of seagrass meadows, we have presented a novel workflow in Google Earth Engine. The approach demonstrates that seagrass mapping is possible over both large spatial (22,840 km^2^) and temporal (time series, n = 22 years) scales, and with high accuracy (overall accuracy =  > 89% in all cases; Table 2). Using Google Earth Engine enabled us to use some 2,570 open-source satellite images across the time series to present a robust temporal analysis of an important and globally declining marine habitat type. We suggest that this workflow could be readily applied in different shallow marine systems both to map and analyse temporal changes in seagrass meadows and other benthic habitats such as coral reefs. The Landsat archive is a highly suitable dataset for such mapping over decadal scales, however, our approach can also be applied to other satellite datasets and when working over shorter timescales. For example, monthly composites, rather than annual composites used here, could be produced to investigate intra-annual changes in seagrass extent. We expect our workflow to be widely applicable over large spatial extents, as the structure of our method is modelled on previous work that has mapped contemporary seagrass area across 113,000 km^2^^16^ and, by implementing regional segmentation, across the full extent of the tropics^18^. The training and validation of such models are crucially important steps which should involve experts with first-hand knowledge and experience of the region mapped. The results from such studies can contribute to national and international scale ecosystem accounting, marine spatial planning, and ecosystem modelling, as well as detailing medium to long term temporal trends in habitat aerial extent.

## Conclusions

Using a novel workflow in GEE, we present the first national scale seagrass-specific map of the Maldives, which showed shallow water seagrass extent in 2021 to be 105 km^2^. Additionally, we have generated a time series of historic national seagrass maps from 2000 to 2021, which reveal a threefold increase in seagrass area – tropical seagrass expansion at the national scale is (to the best of the authors’ knowledge) unique in the context of global seagrass loss. We hypothesize that seagrass success in the Maldives is currently supported by anthropogenic influence in the coastal zone. As the human population continues to grow in tandem with increasing tourism, urbanisation, and fertiliser use, Maldivian seagrasses should still be considered vulnerable to increases in human activity. Continued increases in nutrient levels could create conditions favouring macroalgal growth, resulting in both the loss of seagrass and the potential for coral-macroalgal phase shifts, which would be severely detrimental to the broader reef system. Nonetheless, the threefold increase in seagrass areal extent over the last 20 years highlights that there is not a uniform decline in seagrass meadow extent globally, but rather, at present, Maldivian seagrass beds give cause for ocean optimism. We, therefore, wish to highlight that seagrasses in the Maldives are highly worthy of increased global attention across scientific, commercial, and conservation policy contexts.

## Methods

### Site description

The Maldives is a nation in the central Indian Ocean consisting of 1,192 islands on 26 atolls stretching 870 km north-to-south along the Chagos-Laccadive Ridge^64^ (Fig. 1). The human population of the Maldives has grown considerably over the last 60 years from ~ 92,000 in 1960 to ~ 524,000 in 2022^65^. The climate of the Maldives is tropical, with two distinct monsoonal periods: the southwest monsoon (wet season; Hulhangu in Dhivehi) from May to November and the northeast monsoon (dry season; Iruvai in Dhivehi) from January to March. The Maldivian reef systems as a whole are considered the seventh largest on Earth in area and are comprised of at least 248 species of hard coral belonging to 57 genera^66^. There are at least 8 species of seagrass in the Maldives: *Thalassia hemprichii*, *Thalassodendron ciliatum*, *Halodule pinifolia, Halodule uninervis, Syringodium isoetifolium*, *Cymodocea rotundata*, *Oceana serrulata,* and *Halophila ovalis*.

### Framework for generating the national scale contemporary map

To produce a national contemporary seagrass map for the Maldives, a workflow was adapted using established methods^17,18^. The satellite data used to produce the contemporary maps consisted of Sentinel-2 images which were retrieved and pre-processed in GEE following the standard protocol for aquatic remote sensing of benthic habitats (see full details in supplementary material).

#### Training and validation data design

When implementing a supervised machine learning algorithm, data are used to both train and validate the classification. The training data were independently labelled by 3 of the authors with extensive Maldives fieldwork experience and coastal remote sensing experience through photointerpretation of the Sentinel-2 composite and were further verified by local knowledge and high-resolution Google Earth imagery. Representative training polygons were generated across 3 classes, seagrass, non-seagrass (which included coral reefs, mangroves, sand/rubble, and macroalgal beds), and optical-deep water (ODW), to sample pixel-level data on the spectral, elevation, and slope values of each class. The training data were labelled over the full extent of the Maldives, and across the full range of geomorphological features and water depths, as informed by the bathymetric DEM (10.5 × 10.5 m pixel size) and Allen Coral Atlas geomorphic thematic map (5 × 5 m pixel size)^18^. In total 25,463 training pixels were generated (n = 5761 seagrass; n = 15,433 non-seagrass; n = 4269 ODW; Fig. 1).

Validation of the thematic seagrass maps produced by remote sensing methods is important to quantify the accuracy of the model output^67^. As such, the data used for validation were independent of the training dataset. The validation dataset represented in-situ field surveys collected via SCUBA, snorkel, and low-tide visual methods. The sources included data collated and shared from the Allen Coral Atlas project^20^, Seaview surveys^68^, monitoring by local NGOs, and fieldwork conducted by the authors. In total, 1019 validation points were used at a minimum spacing of 10 m (n = 557 seagrass, n = 462 non-seagrass), sourced from field surveys between 2017 and 2023 (Fig. 1). Misclassification of macroalgae as seagrass and equally seagrass as macroalgae can be a particular problem due to spectral similarities between these two groups. To assess this, we explicitly included 128 validation points with benthic macroalgal cover of > 75%. An error matrix was generated from the validation assessment and the accuracy of the thematic classification was assessed using well-established metrics of mapping accuracy that were: (i) overall accuracy—this metric is an assessment of the proportion of correctly classified validation points; (ii) producer’s accuracy—this metric represents errors of omission (producer’s accuracy = 100%—errors of omission (%)) put simply the degree to which a class is underestimated in the model; and (iii) user’s accuracy—this metric represents errors of commission (user’s accuracy = 100%—errors of commission (%)) or the degree to which a class is overestimated in the model.

####  AI-guided classification

GEE supports the implementation of multiple machine-learning classification algorithms. In this study, a support vector machine (SVM) method was used. This established method can be used to address linear and non-linear problems and works by fitting an optimal hyperplane to the data in asset space^69^. The algorithm fits the hyperplane by maximising the margin from the nearest points to the decision boundary surface. Parameterisation of the model is achieved through adjusting values of cost and gamma. Post-classification, a focalMode() kernel was passed over the raster layer to reduce salt-and-pepper noise.

### Framework for generating national-scale historic seagrass maps

The workflow described in Section “Framework for generating the national scale contemporary map” was used to generate historic seagrass habitat maps to determine shifts in seagrass area and distribution through time. To apply the workflow, an independent composite image was produced for each time point across the time series. There were two key differences between the contemporary and historical map frameworks: a) the data source, and b) the compositing window.

#### Data source and selection

The workflow was applied to the Landsat mission data catalogue. These missions provide satellite data from 1972 to the present and facilitate the analysis of decadal trends in seagrass extent. The data available for this study were limited due to multiple factors. Firstly, the spatial resolution of the Multispectral Scanner (MSS) used onboard Landsat missions 1–3 (operational 1972–1982) was ~ 60 m, which was too coarse for the robust detection of seagrass. Additionally, data available for the Maldives area of interest were mostly classified as tier 2, meaning they did not meet USGS quality control standards for root-mean-square error (< 12 m). Finally, there were significant areas of the Maldives archipelago that were not sampled by Landsat 1–3 during their operational lifespans; gaps in data coverage were also apparent in Landsat 4 data from the MSS and TM sensors (operational 1982–1993). Data from Landsat 5 TM also provided incomplete coverage of the Maldives, north of Malé, between 1990 and 2001. Landsat 7 and 8 data coverage was suitable for compositing from 2000 until 2021, and from 2014 to 2021, respectively.

Subject to the above, data were assembled from over 22 years of monitoring across two satellite missions. Data from 2000 to 2013 were sourced from Landsat 7, and data from 2014 to 2021 were sourced from Landsat 8 (Table 1). Unfortunately, following data filtering, there were insufficient images to generate a composite for the date range 2010–2011.

####  Image compositing

To minimise gaps in spatial coverage, each composite was generated from 2 years of satellite data (from 2000 to 2021, n = 10 composites, or time points, in total). Multi-annual composites are an established means of pre-processing for satellite images, maximising the data available to produce high-quality and low-artefact-composite images whilst maintaining the temporal resolution required to track seagrass change through time^70,71^. Following image compositing, a relative radiometric normalisation was performed across the whole time series using pseudo-invariant features as reference points^72^. Each time series image was numerically matched to the 2020–2021 reference image.

#### Training and validation data design

Training data were manually labelled using the same method applied to the contemporary maps (Table 1). The same independent field data used to validate contemporary maps were used to validate the historic classifications (n = 823 validation points for 2017–2023). In the absence of field data prior to 2017, a fixed pixel approach was used to obtain robust accuracy assessments of the mapping products across the whole time series. This method is established in terrestrial time series validation as limited validation data in time series analysis is common^73–75^. First, a selection of known seagrass and non-seagrass areas from modern validation data were identified and their boundaries were tracked and delineated manually through time. From this selection, areas of consistent seagrass and non-seagrass habitat were identified and used as the boundaries to generate randomly stratified samples. In total, 400 non-seagrass and 400 seagrass validation pixels were used for the fixed pixel accuracy assessments. It is acknowledged that this method may lead to the fixed validation pixels disproportionately representing dense seagrass areas that are more likely to be classified correctly. As such, it is possible that these time series accuracy assessment results may be inflated and should only be used to compare the accuracy among the time series classifications.

###  Data analysis

####  Data collation

The sampling units for this analysis were defined as distinct shallow regions of suitable habitat for seagrass. These areas were identified as polygons from regions of deep water using an unsupervised classification, the Allen Coral Atlas geomorphology product^20^, and data from the Maldives Isles government portal. These regions included reef platforms, lagoons, and other shallow submerged features distinguishable from deep water (Fig. 1 supplementary). For each of these features, data were collated on polygon size (km^2^), seagrass area (km^2^), land area (km^2^), depth (measured in metres; derived from the bathymetry DEM), ruggedness (index^76^; calculated from the DEM as the change in elevation within a 3 × 3 pixel moving sample window), and slope index (calculated from the DEM representing the angle of inclination in degrees). Data on land use and census population size for 2000 and 2017 were obtained from the Maldives Isles government portal. Data on population size were then converted to population density per km^2^.

#### Statistical analysis

For objective 4 we investigated the hypothesis that seagrass presence and occupancy were associated with anthropogenic factors, namely: habitation, land use, and population density. To explore this hypothesis, we tested the following questions: (i) Is seagrass presence more likely on inhabited islands? (ii) Is greater seagrass occupancy associated with certain island land use types and/or more generally with larger (human) population densities? (iii) Across the time series, are increases in seagrass occupancy associated with the magnitude of population growth?

All statistical analysis was conducted in R version 4.1.1 (2021–08-10)^77^. Firstly, a linear regression was used to analyse the relationship between the change in seagrass area across the Maldives and time. The assumption of normality in residuals from the model was confirmed statistically (Shapiro–Wilk, W = 0.9599, p = 0.7847) and homoscedasticity was confirmed with visual inspection.

Logistic regression was used to model the probability of seagrass presence in response to natural and anthropogenic predictors using the R package glmmTMB^78^. An initial model was built incorporating all variables of interest, including measurements of depth (mean), slope (mean), ruggedness (mean), 2017 population density (inhabitants per km^2^), polygon area (km^2^), land area (km^2^), human habitation (binary; 1/0), and a random effect term of atoll ID (categorical with 26 levels). Non-significant terms were dropped sequentially until a minimum adequate model was achieved. The optimal model was selected from the models produced using the Akaike Information Criterion (AIC)^79^. The multicollinearity of predictor variables was tested using the variance inflation factor and was deemed to be low in all cases.

The occupancy of seagrass on inhabited reef platforms with a seagrass presence was explored in response to environmental factors, land use, and population density. As a measure of suitability, occupancy was preferred to raw estimates of extent, as it controlled for the size of each reef platform (i.e. the maximum size of each reef platform/polygon) whilst also disregarding (unsuitable) land area. Or intuitively, the proportion of suitable habitat area on each reef platform that was occupied by seagrass. Seagrass occupancy was calculated as:

$${\text{Seagrass occupancy }}\left( \% \right)\, = \,\left( {{\text{seagrass area }}\left( {{\text{km}}^{{2}} } \right){/ }\left( {{\text{polygon area }}\left( {{\text{km}}^{{2}} } \right){-\!\!-}{\text{land area }}\left( {{\text{km}}^{{2}} } \right)} \right)} \right) \, *{ 1}00$$

A generalised mixed effects model with a negative binomial link was fitted to the data using the package glmmTMB ^78^. The residuals from the model were not zero-inflated but did indicate overdispersion, tested using the DHARMa package^80^. Before analysis, all continuous predictor variables were standardised. Land uses were coded as a categorical variable with 7 categories: 1 = domestic inhabitation; 2 = resort; 3 = agricultural; 4 = airport; 5 = industrial; 6 = fisheries; 7 = aquaculture. All other factors were compared to category 1 = domestic inhabitation. Where reef platforms had multiple land used types, the majority land use in terms of area was recorded. Categories with too few data points were not included in the model, these were airport (n = 6), fisheries (n = 2), and aquaculture (n = 4). A full model was initially built with atoll ID included as a random effect. Non-significant terms were dropped sequentially until a minimum adequate model was achieved. The optimal model was chosen from the lowest AIC.

Finally, the effect of population growth—used as a proxy for anthropogenic pressure—on seagrass area increase over the time series was tested. Seagrass gains were calculated from occupancy increases from 2000 to 2001 until 2020 to 2021. Human population increases were calculated from available census data from 2000 and 2017. A generalised linear Poisson model with a logit link was used. The model did not exhibit overdispersion or zero inflation when tested using the DHARMa package^80^. The human population increase was used as a single predictor of seagrass area increase and atoll ID was included as a random effect.

### Supplementary Information

Supplementary Information.

## Data Availability

The datasets generated during and analysed during the current study are available at DOI: 10.5281/zenodo.11144240. Please contact MF for any further data or code requests.
