# Supplementary material for: Rapid seagrass meadow expansion in an Indian Ocean bright spot
Source: Sci Rep. 2024 May 13;14:10879. doi: 10.1038/s41598-024-61088-1 (PMC11091126; doi:10.1038/s41598-024-61088-1)
Supplement: Supplementary file 1 — Supplementary Information. [file 41598_2024_61088_MOESM1_ESM.docx]

**Rapid seagrass meadow expansion in an Indian Ocean bright spot**

Authors: Matthew Floyd^1*^, Holly K. East^1^, Dimosthenis Traganos^2^, Azim Musthag^3^, James Guest^4^, Aminath S. Hashim^5^, Vivienne Evans^6^, Stephanie Helber^1^, Richard K. F. Unsworth^7^, Andrew J. Suggitt^1^

^1^Department of Geography and Environmental Sciences, Faculty of Engineering and Environment, Northumbria University, Newcastle upon Tyne, NE1 8ST, UK

^2^German Aerospace Centre (DLR), Remote Sensing Technology Institute, Berlin, 12489, Germany

^3^Small Island Research Group, Faresmaathoda, 10780, Maldives

^4^School of Natural and Environmental Sciences, Newcastle University, Newcastle upon Tyne, NE1 7RU, UK

^5^Blue Marine Foundation, M. Beach Side, Handhuvaree Hingun, Malé, 20285, Maldives

^6^Blue Marine Foundation, Somerset House, Strand, London, WC2R 1LA, UK

^7^Seagrass Ecosystem Research Group, Faculty of Science and Engineering, Swansea University, Swansea, SA2 8PP, Wales, UK

**Full details of satellite data pre-processing steps**

1. **Data selection.** Pixels from Sentinel-2 level-1C top-of-atmosphere (TOA) images (100 km x 100 km extent; 10 m spatial resolution) were used as the starting point for analysis. The data used were from 6 input bands: B2 – blue, B3 – green, B4 – red, B8 – near-infrared, B11 – short-wave infrared, and bitmask band QA60 (Traganos et al., 2018). All data were hosted within GEE as an image collection.
2. **Data filtering.** The Sentinel-2 image collection was filtered to reduce the computational load of the subsequent pre-processing steps and to discard low-quality images. Firstly, a cloud pixel filter was applied to remove images containing pixels with >50% cloud cover following a series of trials that set varying limits of cloud cover, to optimise both the number of images in the collection and image quality. Secondly, the image collection was filtered to the date range 01.01.2021-31.12.2021. This process yielded a filtered image collection of 881 Sentinel-2 TOA images.
3. **Cloud mask.** To remove pixels containing opaque and cirrus cloud cover, a cloud mask was applied to the data in the filtered image collection. This step utilised the Sentinel-2 QA60 band to develop a bitmask by selecting pixels with clear, non-cloudy, conditions (Traganos et al., 2018).
4. **Image composite**. Annual compositing was used to create a cloud-free image from the filtered and cloud-masked image collection (Ahmed et al., 2020; Buchner et al., 2020; Mahdianpari et al., 2020). Image compositing is an artefact reduction technique used to generate a single multispectral pseudo-image from image collections. The resulting image contained pixels that were in the first 20^th^ percentile of the pixels from the filtered and masked image collection (Blume et al., 2023; Traganos et al., 2022, 2018). In this way, data from all 881 Sentinel-2 TOA images were fed into generating the final pseudo-image.
5. **Atmospheric correction**. To remove atmospheric interference in the composite, a dark pixel subtraction was performed (Traganos et al., 2022, 2018; Vanderstraete et al., 2006; Wicaksono et al., 2017). The dark pixels were sampled from optically deep water within atoll lagoons, >40 m depth (Traganos et al., 2018), identified from a bathymetric Digital Elevation Model (DEM) (Rasheed et al., 2021). This method extracted the mean and standard deviation reflectance values from dark pixels where path radiance and noise constitute most of the signal. These values were then subtracted from the composite image bands to minimise atmospheric artefacts in the image as a whole (Green et al., 2000; Vanderstraete et al., 2006).
6. **Water column correction**. To account for the effects of light attenuation in water, the Depth Invariance Index (DII) was calculated for three visible band combinations (Green et al., 2000; Poursanidis et al., 2021) for addition to the feature space dataset.
7. **Addition of auxiliary bands**. To increase the data available to the classifier, a Maldives bathymetric DEM (Rasheed et al., 2021; spatial resolution = 10.5 m) was included as an auxiliary dataset covering 1°S to 7.2°N and 72°E to 74°E. This dataset was joined with the spectral composite bands, along with a calculated value of slope (derived from the DEM, representing the angle of inclination in degrees) (Poursanidis et al., 2021). The subsequent feature space contained 6 spectral bands, 3 DII bands, bathymetry, and slope. All data were then normalised to a range between 0 and 1 before use in the machine learning classifier.
8. **Area of Interest clip**. The composite image was clipped to the area of interest using a Maldives atoll shapefile ([humdata.org](https://data.humdata.org/), 2022), as provided by the Government of the Maldives. This dataset was visually inspected and manually amended to correct areas of misalignment between satellite imagery and the atoll polygons and to ensure that all areas of interest were included in all polygons. The resulting area of interest was 22,840 km^2^.
9. **Land cover mask**. Areas of land were not of interest in this study, so a land mask was developed to remove these pixels from the composite image. The land mask was developed by calculating the Modified Normalised Difference Water Index (MNDWI) using the green and short-wave infrared bands (*MNDWI = (GREEN-SWIR)/(GREEN+SWIR)*; Ahmed et al., 2020). A binary classification of land and water was then undertaken, using a threshold value of 0.2, and applied as a mask. A land mask developed from the composite image from each timeframe of interest was used to account for the dynamic nature of reef island land (Carruthers et al., 2023).
10. **Deep water mask.** Finally, a deep-water mask was applied using the bathymetry DEM to remove areas where seagrass was unlikely to be found and unlikely to be mapped with accuracy using this approach. The mask used in this study removed areas deeper than 20 metres, firstly, because validation data were unavailable for deeper sites, secondly, because it is difficult to obtain accurate benthic signals using satellites below 20 metres (Traganos and Reinartz, 2018). We acknowledge that although *T. ciliatum* most commonly occurs up to 17 metres (Short et al., 2010), it is recorded below 20 metres in the Western Indian Ocean (Esteban et al., 2018).


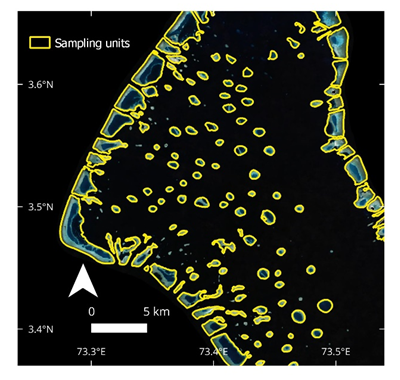


*Supplementary figure 1. Example of distribution of distinct reef platforms (i.e. shallow areas) in the Maldives used as sampling units for statistical analysis.*

*Supplementary table 1. Contemporary seagrass area across Maldivian atolls.
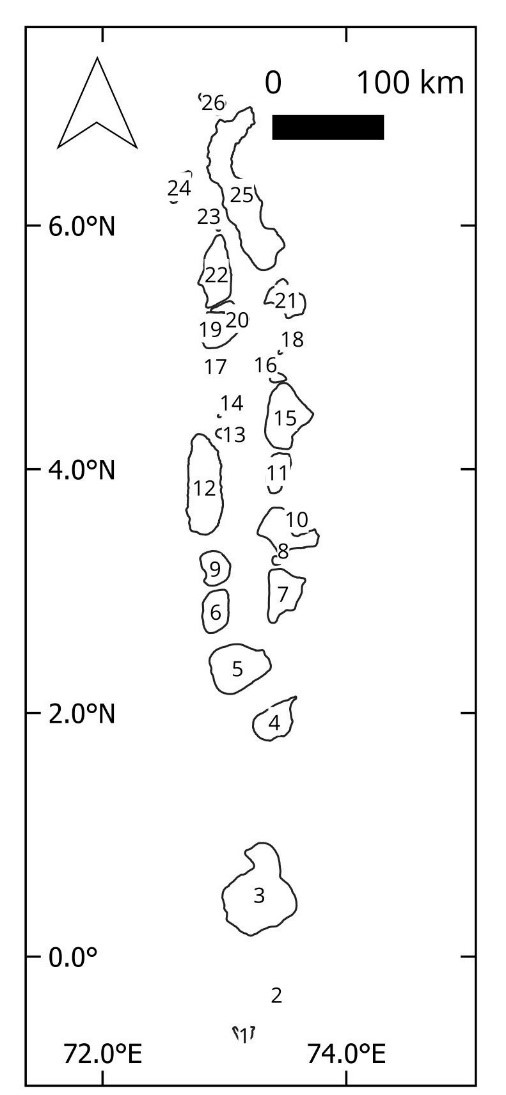
*

| Atoll ID | Atoll name | Seagrass area (km2) |
| --- | --- | --- |
| 1 | Addu | 5.0704 |
| 2 | Fuvahmulah | 0.0439 |
| 3 | Huvadhoo | 29.2924 |
| 4 | Laamu | 21.8974 |
| 5 | Thaa | 3.9931 |
| 6 | Dhaalu | 0.1072 |
| 7 | Meemu | 19.4032 |
| 8 | Vattaru | 0.0014 |
| 9 | Faafu | 0.0964 |
| 10 | Vaavu | 0.0312 |
| 11 | South Male' (Kaafu) | 1.5463 |
| 12 | Ari | 1.1141 |
| 13 | Rasdhoo | 0.0032 |
| 14 | Thoddoo | 0.0098 |
| 15 | North Male' (Kaafu) | 0.9568 |
| 16 | Gaafaru (Kaafu) | 0.1828 |
| 17 | Goidhoo (Baa) | 3.3982 |
| 18 | Kaashidhoo (Kaafu) | 1.7157 |
| 19 | Baa | 0.8079 |
| 20 | Kudarikilu (Baa) | 0.0046 |
| 21 | Lhaviyani | 7.7383 |
| 22 | Raa | 0.2917 |
| 23 | Alifushi (Raa) | 0.1337 |
| 24 | Haa Dhaalu | 1.5818 |
| 25 | Shaviyani | 3.1889 |
| 26 | Haa Alifu | 2.1781 |
| Total |  | 104.7885 |

*Supplementary table 2. Model output tables*

| Linear model of seagrass area change over the time series | | | | |
| --- | --- | --- | --- | --- |
|  | estimate | standard error | z value | p |
| intercept | -9183.94 | 824.68 | -11.14 | <0.001 |
| year | 4.60 | 0.41 | 11.22 | <0.001 |

| Logistic regression of seagrass presence/ absence | | | | |
| --- | --- | --- | --- | --- |
|  | estimate | standard error | z value | p |
| intercept | 1.64 | 0.42 | 3.87 | 0.00 |
| depth mean | 0.19 | 0.03 | 6.50 | <0.001 |
| ruggedness mean | -0.40 | 0.05 | -8.64 | <0.001 |
| habitation | 1.49 | 0.16 | 9.48 | <0.001 |

| Generalised linear model of seagrass occupancy | | | | |
| --- | --- | --- | --- | --- |
|  | estimate | standard error | z value | p |
| intercept | -231.68 | 217.96 | -1.06 | 0.29 |
| depth mean | 1.18 | 0.48 | 2.48 | <0.05 |
| ruggedness mean | -1.99 | 0.36 | -5.55 | <0.001 |
| slope mean | 235.39 | 218.09 | 1.08 | 0.28 |
| resort | -0.65 | 41389.00 | -1.57 | 0.12 |
| agricultural | -1.62 | 0.36 | -4.57 | <0.001 |
| industrial | -0.78 | 0.40 | -1.98 | <0.05 |
| population density 2017 | -0.03 | 0.06 | -0.44 | 0.66 |

| Generalised linear model of seagrass area change | | | | |
| --- | --- | --- | --- | --- |
|  | estimate | standard error | z value | p |
| intercept | 1.60 | 0.23 | 7.01 | <0.001 |
| platform population change | 0.00003961 | 0.00005903 | 0.67 | 0.50 |

**References**

Ahmed, A.F., Mutua, F.N., Kenduiywo, B.K., 2020. Monitoring benthic habitats using Lyzenga model features from Landsat multi-temporal images in Google Earth Engine. Model. Earth Syst. Environ. <https://doi.org/10.1007/s40808-020-00960-1>

Blume, A., Pertiwi, A.P., Lee, C.B., Traganos, D., 2023. Bahamian seagrass extent and blue carbon accounting using Earth observation. Front. Mar. Sci.

Buchner, J., Yin, H., Frantz, D., Kuemmerle, T., Askerov, E., Bakuradze, T., Bleyhl, B., Elizbarashvili, N., Komarova, A., Lewińska, K.E., Rizayeva, A., Sayadyan, H., Tan, B., Tepanosyan, G., Zazanashvili, N., Radeloff, V.C., 2020. Land-cover change in the Caucasus Mountains since 1987 based on the topographic correction of multi-temporal Landsat composites. Remote Sens. Environ. 248, 111967. <https://doi.org/https://doi.org/10.1016/j.rse.2020.111967>

Carruthers, L., East, H., Ersek, V., Suggitt, A., Campbell, M., Lee, K., Naylor, V., Scurrah, D., Taylor, L., 2023. Coral reef island shoreline change and the dynamic response of the freshwater lens, Huvadhoo Atoll, Maldives . Front. Mar. Sci. .

Esteban, N., Unsworth, R.K.F., Gourlay, J.B.Q., Hays, G.C., 2018. The discovery of deep-water seagrass meadows in a pristine Indian Ocean wilderness revealed by tracking green turtles. Mar. Pollut. Bull. 134, 99–105. <https://doi.org/https://doi.org/10.1016/j.marpolbul.2018.03.018>

Green, E., Mumby, P., Edwards, A., Clark, C., 2000. Remote Sensing Handbook for Tropical Coastal Management.

Mahdianpari, M., Jafarzadeh, H., Granger, J.E., Mohammadimanesh, F., Brisco, B., Salehi, B., Homayouni, S., Weng, Q., 2020. A large-scale change monitoring of wetlands using time series Landsat imagery on Google Earth Engine: a case study in Newfoundland. GIScience Remote Sens. 57, 1102–1124. <https://doi.org/10.1080/15481603.2020.1846948>

Poursanidis, D., Traganos, D., Teixeira, L., Shapiro, A., Muaves, L., 2021. Cloud-native seascape mapping of Mozambique’s Quirimbas National Park with Sentinel-2. Remote Sens. Ecol. Conserv. 7, 275–291. <https://doi.org/https://doi.org/10.1002/rse2.187>

Rasheed, S., Warder, S., Plancherel, Y., Piggott, M., 2021. An Improved Gridded Bathymetric Data Set and Tidal Model for the Maldives Archipelago. Earth Sp. Sci. 8. <https://doi.org/10.1029/2020EA001207>

Short, F.T., Coles, R., Waycott, M., Bujang, J.S., Fortes, M., Prathep, A., Kamal, A.H.M., Jagtap, T.G., Bandeira, S., Freeman, A.S., Erftemeijer, P.L.A., La Nafie, Y.A., Vergara, S.G., Calumpong, H.P., Makm, I., 2010. Thalassodendron ciliatum. <https://doi.org/http://dx.doi.org/10.2305/IUCN.UK.2010-3.RLTS.T173375A7002484.en>

Traganos, D., Aggarwal, B., Poursanidis, D., Topouzelis, K., Chrysoulakis, N., Reinartz, P., 2018. Towards Global-Scale Seagrass Mapping and Monitoring Using Sentinel-2 on Google Earth Engine: The Case Study of the Aegean and Ionian Seas. Remote Sens. . <https://doi.org/10.3390/rs10081227>

Traganos, D., Lee, C.B., Blume, A., Poursanidis, D., Čižmek, H., Deter, J., Mačić, V., Montefalcone, M., Pergent, G., Pergent-Martini, C., Ricart, A.M., Reinartz, P., 2022. Spatially Explicit Seagrass Extent Mapping Across the Entire Mediterranean . Front. Mar. Sci. .

Traganos, D., Reinartz, P., 2018. Mapping Mediterranean seagrasses with Sentinel-2 imagery. Mar. Pollut. Bull. 134, 197–209. <https://doi.org/https://doi.org/10.1016/j.marpolbul.2017.06.075>

Vanderstraete, T., Goossens, R., Ghabour, T.K., 2006. The use of multi‐temporal Landsat images for the change detection of the coastal zone near Hurghada, Egypt. Int. J. Remote Sens. 27, 3645–3655. <https://doi.org/10.1080/01431160500500342>

Wicaksono, P., Salivian Wisnu Kumara, I., Kamal, M., Afif Fauzan, M., Zhafarina, Z., Agus Nurswantoro, D., Noviaris Yogyantoro, R., 2017. Multispectral Resampling of Seagrass Species Spectra: WorldView-2, Quickbird, Sentinel-2A, ASTAB. IOP Conf. Ser. Earth Environ. Sci. 98, 12039. <https://doi.org/10.1088/1755-1315/98/1/012039>
